# Mr-AbaA Regulates Conidiation by Interacting with the Promoter Regions of Both *Mr-veA* and *Mr-wetA* in Metarhizium robertsii

**DOI:** 10.1128/Spectrum.00823-21

**Published:** 2021-09-08

**Authors:** Hao Wu, Youmin Tong, Rong Zhou, Yulong Wang, Zhangxun Wang, Ting Ding, Bo Huang

**Affiliations:** a Anhui Provincial Key Laboratory of Microbial Pest Control, Anhui Agricultural University, Hefei, China; b School of Plant Protection, Anhui Agricultural University, Hefei, China; University of Molise

**Keywords:** conidiation, *Metarhizium robertsii*, transcription factor, regulation mechanism

## Abstract

Conidiation is a pivotal strategy for fungi to resist adverse environments and disperse to new habitats, which is especially important for entomopathogenic fungi whose conidia are infective as fungal pesticide propagules. However, the molecular mechanism for regulating conidiation in entomopathogenic fungi is not fully understood. Here, we characterized the regulatory mechanism of the key developmental transcription factor Mr-AbaA. Bioinformatic analysis, transcriptional profiles, and subcellular localization of *Mr-abaA* indicated that AbaA functioned as a transcription factor in the conidiophore development and conidium stages. Microscopic examination showed that the null mutant of *Mr-abaA* differentiated into defective phialides to produce an abacus structure instead of conidia. Loss of *Mr-abaA* resulted in the inhibition of submerged blastospore separation *in vitro*. Moreover, yeast (Saccharomyces cerevisiae) one-hybrid assays of interactions between genes and deletion of *Mr-veA* showed that Mr-AbaA regulates conidiation by interacting with the promoter regions of *Mr-veA* and *Mr-wetA*. These results demonstrate that Mr-AbaA positively regulates conidiation in Metarhizium robertsii by regulating the *velvet* family ortholog gene *Mr-veA* and contributes to the separation of blastospores in submerged culture.

**IMPORTANCE** Metarhizium robertsii is an emerging model entomopathogenic fungus for developing biopesticides; therefore, a comprehensive understanding of its conidiation is very important for its application. In this study, we revealed that the transcription factor Mr-AbaA is involved in the control of aerial conidiation and blastospore separation in submerged culture. Further yeast one-hybrid assays demonstrated that Mr-AbaA interacts with the promoter regions of *Mr-veA* and *Mr-wetA*, which code for proteins involved in the control of conidiation. This finding provides new insight into the regulation of the conidiation of this important entomopathogenic fungi.

## INTRODUCTION

Many species of entomopathogenic fungi play crucial roles in worldwide agroforestry pest management. As an emerging model of entomopathogenic fungi, Metarhizium spp. have been developed for biopesticides instead of chemical insecticides because of the absence of detrimental environmental effects and the ease of mass production (1). Conidia serve as the main units of environmental dispersal, invasion, and proliferation for Metarhizium robertsii. Moreover, conidia are the main components of fungal pesticides. However, the low yield of conidia and their sensitivity to environmental conditions have limited the large-scale application of M. robertsii (2). Understanding the molecular mechanisms controlling conidium production and increasing conidium yield and resistance to stress for M. robertsii by genetic manipulation are essential for commercial development.

Asexual sporulation is the most common reproductive strategy in filamentous fungi. Conidiogenesis is genetically programmed, and distinct gene sets are responsible for the progression of each phase (3). In the genetic regulation of asexual development in filamentous fungi, many researchers have extensively studied the model fungi Neurospora crassa and Aspergillus nidulans (4, 5). Many regulatory genes, including central regulators, negative regulators, upstream activators, *velvet* regulators, and light-responsive genes, are involved in conidiogenesis, but a central regulatory pathway comprising the three key regulators *brlA*, *abaA*, and *wetA* plays a crucial role in asexual development (6, 7). Overall, the key regulators *abaA* and *wetA* are well conserved among most filamentous fungi (6, 8, 9). The C_2_H_2_ zinc finger transcription factor *brlA* governs the initiation of conidiophore development and subsequently activates *abaA* during the middle stages of conidiophore development (10). Then, WetA, the expression of which is induced by AbaA in the late stage of conidiation, activates the expression of proteins or enzymes involved in the synthesis of conidium wall components, which is required for conidial maturation (11, 12).

The *abaA* gene encodes a developmental transcription factor with an ATTS/TEA DNA-binding domain that is required for the differentiation of phialides during the middle stages of A. nidulans conidiation (3, 13–15). In A. nidulans and Aspergillus fumigatus, loss of *abaA* resulted in the formation of abnormal metulae and phialides that produce long chains of cells that appear like beads on a string, as in an abacus (16, 17). Similarly, deletion of *abaA* in *Talaromyces* (formerly *Penicillium*) *marneffei* blocks asexual development and results in aberrant conidiophores with reiterated terminal cells (18). Similar phenotypes were seen in other filamentous fungi, such as Fusarium graminearum, Penicillium digitatum, and Beauveria bassiana (19–21). In addition, *abaA* was reported to govern dimorphic growth in T. marneffei and B. bassiana (18, 21). As mentioned above, *wetA* is activated by AbaA to complete conidiation. Aside from *wetA* expression, AbaA also positively regulates the transcript levels of two *velvet* family genes, *velB* and *vosA*, during conidiogenesis and directly binds to the promoter regions of those genes in A. nidulans (22). The *velB* and *vosA* genes not only are involved in asexual development and conidiogenesis but also play interdependent roles in trehalose biogenesis, conidial viability, and controlled conidial germination (23–25).

The process of asexual reproduction in M. robertsii is divided into the vegetative growth phase and the development phase. The formation of conidia takes place in the development phase and starts with the formation of conidiophores branching repeatedly at broad angles. Afterward, the tip of the conidiophore gives rise to clavate or cylindrical phialides in dense hymenia. Finally, repeated mitotic divisions occur in phialides to produce conidia in long chains. In M. robertsii, a conserved central regulatory pathway consisting of Mr-BrlA, Mr-AbaA, and Mr-WetA was identified; Mr-BlrA regulates *Mr-abaA*, which in turn activates *Mr-wetA* during conidiation (26). Deletion of *Mr-brlA* and *Mr-abaA* resulted in inhibition of conidium production, while deletion of *Mr-wetA* resulted in reduced conidial yields (26). Similar phenotypes were seen in B. bassiana. Loss of *brlA* or *abaA* resulted in inhibition of aerial conidiation, while knockout mutants of *wetA* and *vosA* lost most of their conidiation capacities (21, 27). Based on a previous framework from the study of M. robertsii conidiation, we primarily focused on the characterization and regulatory mechanism of the key developmental transcription factor Mr-AbaA.

## RESULTS

### Characteristics and deletion of *Mr-abaA*.

**FIG 1:**
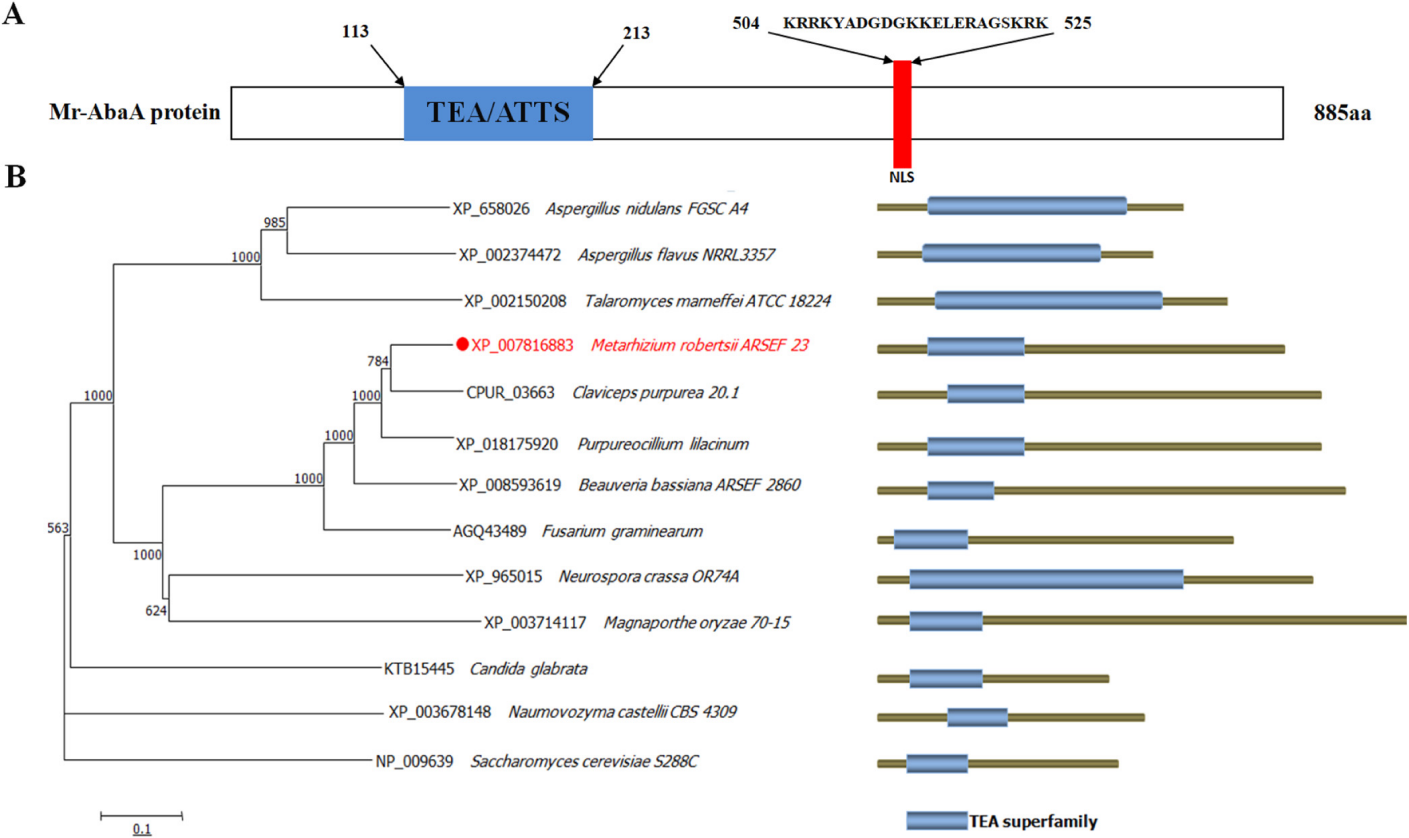
Bioinformatic analysis of *Mr-abaA*. (A) Structure domain analysis of the *Mr-abaA* protein. TEA, ATTS/TEA domain family. (B) Phylogenetic tree analysis of *abaA* orthologs from several fungi. The labels on the right display the NCBI accession numbers and the fungal species.

A previous analysis identified M. robertsii MAA-00694 (*Mr-abaA*) as a homolog of A. nidulans *abaA* by BLASTP (26). The open reading frame (ORF) of this gene consists of 2,658 nucleotides, contains two introns and three exons, and encodes a protein of 885 amino acids. Conserved functional domain analysis showed that it conserves an ATTS/TEA family domain (NCBI accession number pfam01285: https://www.ncbi.nlm.nih.gov/Structure/cdd/cddsrv.cgi?uid=pfam01285). A nuclear localization signal (NLS) motif was predicted in the C terminus of Mr-AbaA (residues 504 to 525) at NLStradamus (http://www.moseslab.csb.utoronto.ca/NLStradamus) (Fig. 1A). Sequence alignment analysis revealed a much higher sequence identity of *Mr-abaA* to the orthologs of Claviceps purpurea (82%) and Purpureocillium lilacinum (79%) than to the orthologs of these species. Further phylogenetic tree analysis indicated that *Mr-abaA* is also relatively closer to C. purpurea and P. lilacinum than to other fungi (Fig. 1B). Moreover, M. robertsii, C. purpurea, and P. lilacinum all belong to the family Clavicipitaceae.

To assess the biological functions of *Mr-abaA* in M. robertsii, the targeted gene knockout vector (pDHt-SK-*bar*-*Mr-abaA*) was inserted into the wild-type (WT) strain to construct the Δ*Mr-abaA* deletion mutant via agrobacterium-mediated homologous recombination. The confirmation of gene deletion by PCR and reverse transcription (RT)-PCR is presented in Fig. S1 in the supplemental material.

### Transcriptional profiles and subcellular localization of Mr-AbaA.

**FIG 2:**
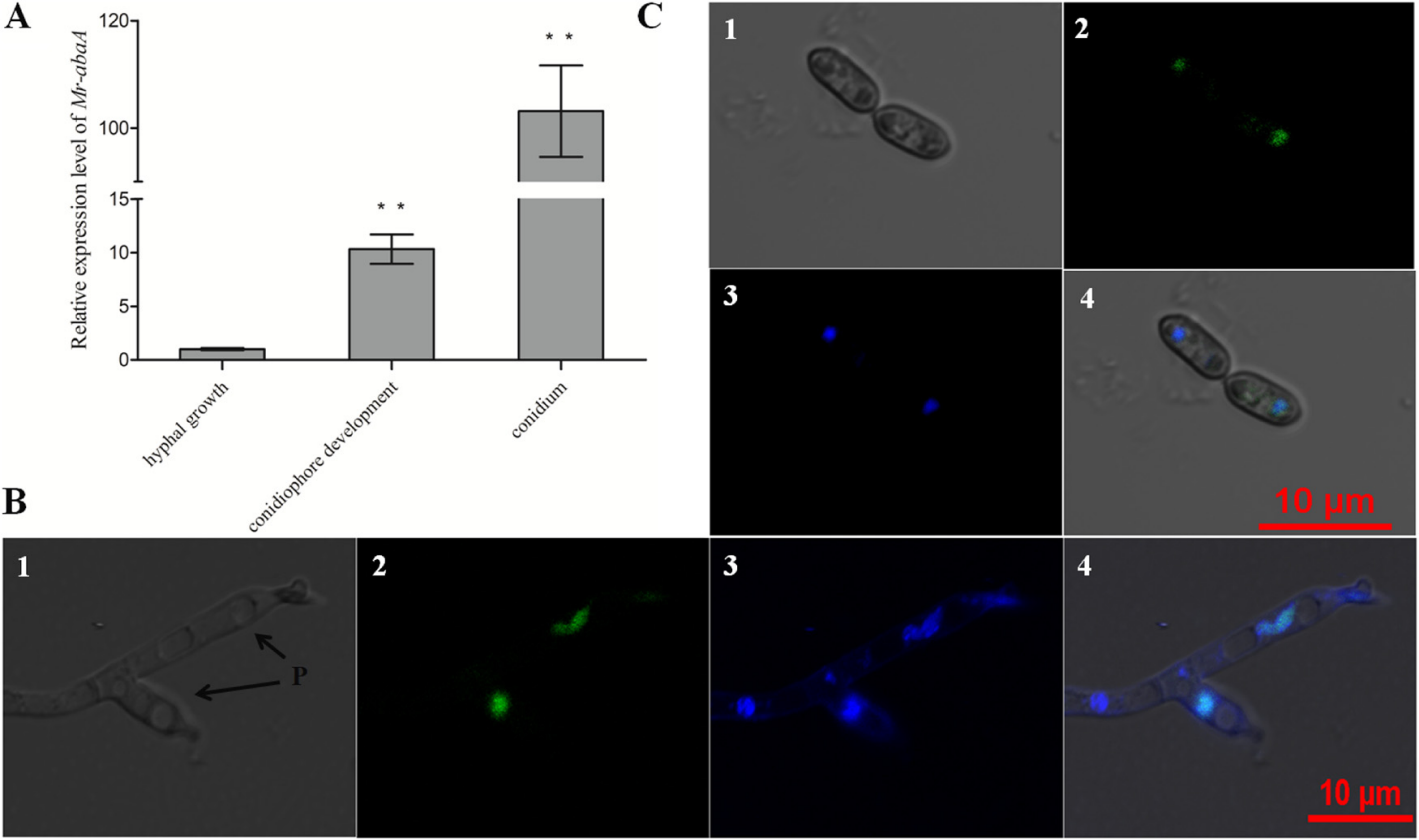
Transcriptional profiles and subcellular localization of Mr-AbaA. (A) Relative transcript levels of *Mr-abaA* in the WT cultures in three different developmental stages, compared with the standard level during hyphal growth. **, *P < *0.01, Tukey’s HSD tests. (B and C) Subcellular localization of AbaA::GFP fusion protein expressed in the conidiophore development (B) and conidium (C) stages. Nuclei were stained with DAPI. Brightfield, expressed (green), DAPI-stained (blue), and merged views of the same field are numbered 1, 2, 3, and 4, respectively. P, phialides. Error bars in panel A indicate standard deviations of the means from three independent replicates.

Transcriptional profiles of *Mr-abaA* were monitored in three different developmental stages, including hyphal growth, conidiophore development, and the conidium stage (Fig. 2A). Compared with the standard level in hyphal growth, the *Mr-abaA* transcript level was sharply increased in conidiophore development and the conidium stage (Tukey’s honestly significant difference [HSD] tests, *P < *0.01 [*n *= 3]). Notably, a significant elevation to approximately 100-fold greater *Mr-abaA* transcript levels was detected in the conidium stage, compared with the hyphal growth stage. These data suggest that *Mr-abaA* may function in the conidium and conidiophore development stages.

The fungal cells in three different developmental stages were visualized for subcellular localization of enhanced green fluorescent protein (EGFP)-tagged Mr-AbaA fusion protein expressed in the WT strain, stained with 4′,6-diamidino-2-phenylindole dihydrochloride (DAPI). The merged image of EGFP and DAPI staining showed that Mr-AbaA localized to the nucleus of phialides in the conidiophore development stage (Fig. 2B) and the nucleus of 10-day-old conidium (Fig. 2C). However, the green signal was not detected in the hyphal growth stage. Thus, this observation from subcellular localization analysis is consistent with the transcriptional profiles of *Mr-abaA*. In addition, these results imply the possibility that *Mr-abaA* acts as a transcription factor that functions in conidium and conidiophore development stages.

### *Mr-abaA* is indispensable for aerial conidiation but does not affect hyphal growth.

For radial growth, Δ*Mr-abaA* showed similar colony sizes in potato-dextrose agar (PDA), Sabouraud dextrose agar with yeast (SDAY) medium, and one-quarter-strength SDAY (1/4SDAY) medium, compared with the WT strain. These data indicated that colony growth was not affected by *Mr-abaA* deletion (see Fig. S2A).

**FIG 3:**
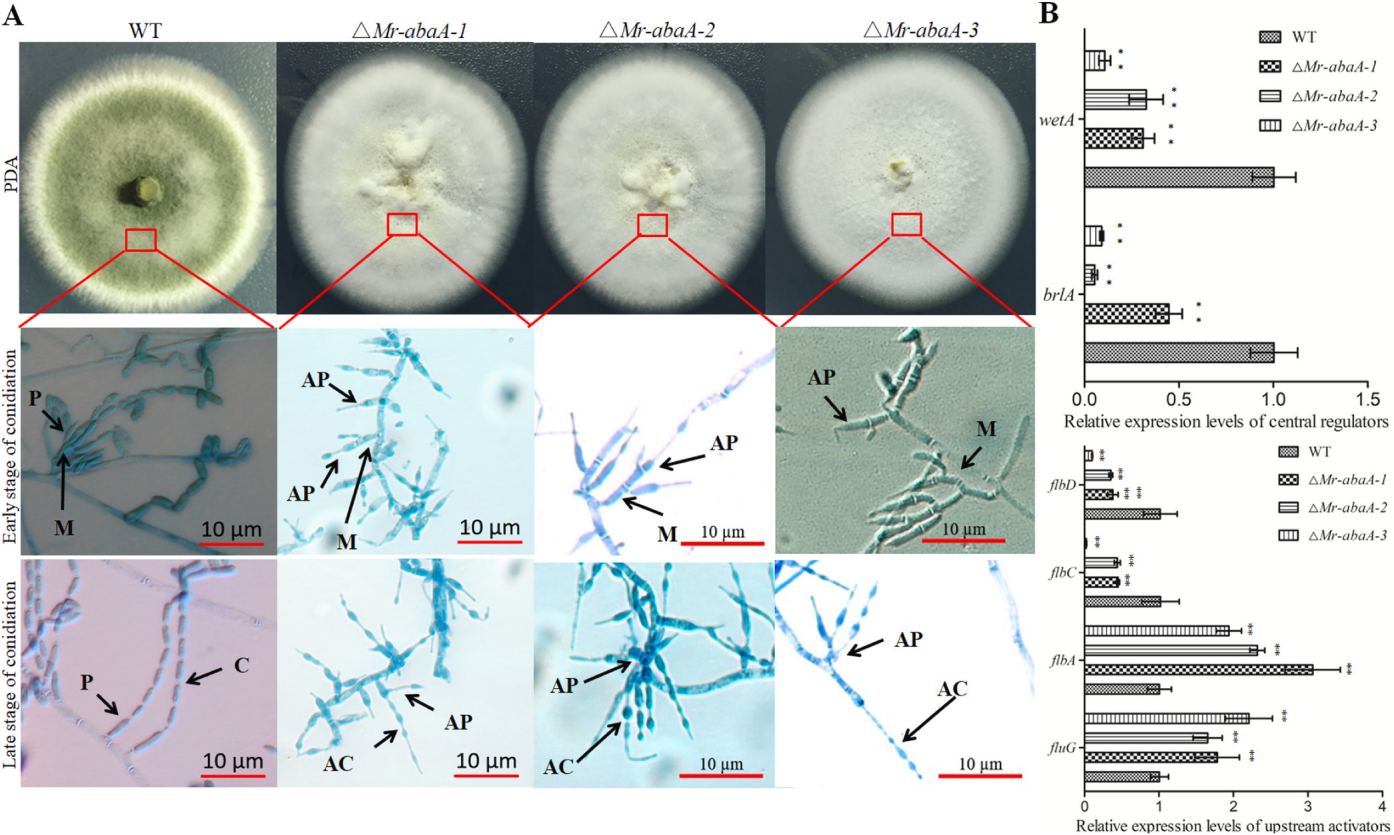
Phenotypic analysis of the WT and Δ*Mr-abaA* strains. (A) Three independent Δ*Mr-abaA* strains showed a change in colony color. To observe the conidiophores on the aerial hyphae, WT and Δ*Mr-abaA* cells were grown on PDA plates and sampled at 2.5 dpi (early stage of conidiation) and 10 dpi (late stage of conidiation). WT conidiophores had metulae (M), phialides (P), and conidia (C), whereas Δ*Mr-abaA* conidiophores had metulae (M), abnormal phialides (AP), and abacus aberrant conidia (AC). (B) qRT-PCR analysis of the expression levels of conidiation-related genes among WT and null mutant clones. **, *P < *0.01, Tukey’s HSD tests. Error bars in panel B indicate standard deviations of the means from three independent replicates.

Deletion of *Mr-abaA* resulted in inhibition of aerial conidiation, and we were not able to obtain a *Mr-abaA* complementation strain using conidia as recipients. Therefore, we present assay data from three independent mutants. The gene disruption mutants and the WT strain were cultivated on different media, and their phenotypes were observed and compared. WT colonies were initially white, usually became yellow during the early development of conidia, and then became greenish as the conidia matured on PDA and 1/4 SDAY medium. However, the colony pigmentation of the Δ*Mr-abaA* mutant was always white (Fig. 3A). Microscopically, the abacus aberrant conidia from the Δ*Mr-abaA* mutant are responsible for the changed colony color, compared with the WT strain. Microscopic observation showed that the Δ*Mr-abaA* mutant produces morphologically WT metulae. However, its scattered phialides produce short abacus aberrant conidia rather than cylindrical conidia (Fig. 3A). Thus, the loss of *Mr-abaA* interrupted the differentiation of phialides.

*Mr-abaA* was indispensable for completing conidiation under aerial conditions. To investigate the functions of *Mr-abaA* in conidiation, the expression levels of conidiation-related genes in filamentous fungi were determined by quantitative RT-PCR (qRT-PCR). The results showed that the relative expression levels of genes such as upstream activators *flbC* and *flbD* or central regulators *brlA* and *wetA* were significantly downregulated, while the relative expression of others such as *fluG* and *flbA* were significantly upregulated in the Δ*Mr-abaA* mutant, compared with the WT strain (Tukey’s HSD tests, *P < *0.01 [*n *= 3]) (Fig. 3B) (6, 28–31). Thus, deletion of *Mr-abaA* resulted in significantly altered expression levels for the conidiation-related genes analyzed.

**FIG 4:**
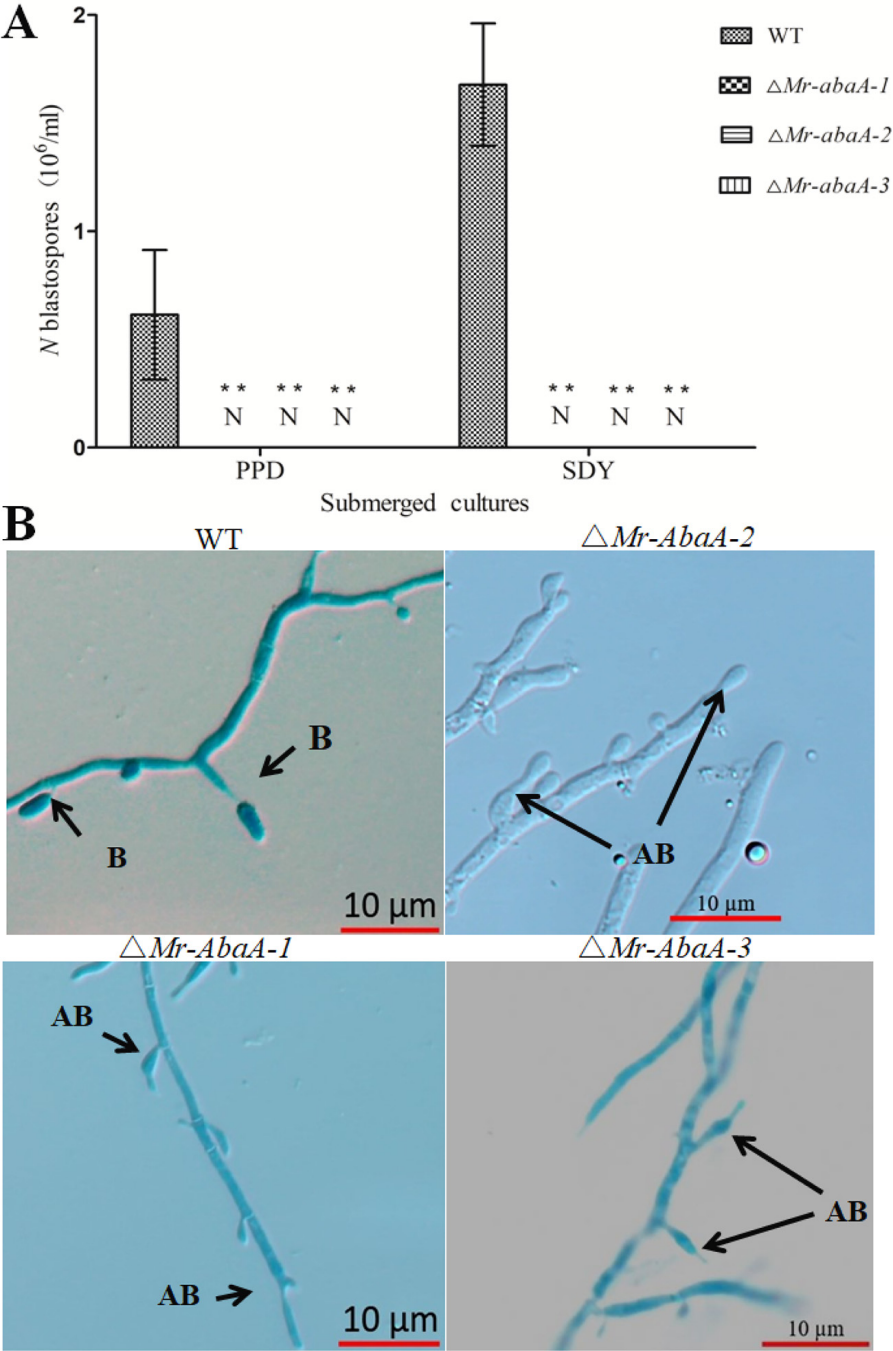
Indispensable roles of *Mr-abaA* in blastospore separation. (A) Blastospore yields were quantified from 4-day-old submerged cultures of WT and Δ*Mr-abaA* strains in SDB medium and PPD medium, respectively. No detectable (N) blastospores were observed for the Δ*Mr-abaA* strain. **, *P < *0.01, Tukey’s HSD tests. (B) In submerged broth *in vitro*, the WT strain formed blastospores (B), while the Δ*Mr-abaA* strain generated abnormal blastospores (AB). Error bars in panel A indicate standard deviations of the means from three independent replicates.

Knockout of *Mr-abaA* resulted in complete interruption of blastospore separation in submerged cultures. After 3 days of culture in Sabouraud dextrose broth (SDB) and potato-peptone-dextrose (PPD) broths, the WT strain generated 0.61(±0.3; *n* = 6) × 10^6^ spores/ml and 1.68(±0.28; *n* = 6) × 10^6^ spores/ml, respectively (Fig. 4A). However, microscopic examination demonstrated that the Δ*Mr-abaA* mutant generated normal conidiogenous cells but blastospores were tightly connected to conidiogenous cells and did not separate from those in SDB (Fig. 4B).

### *Mr-abaA* is important for heat tolerance.

**FIG 5:**
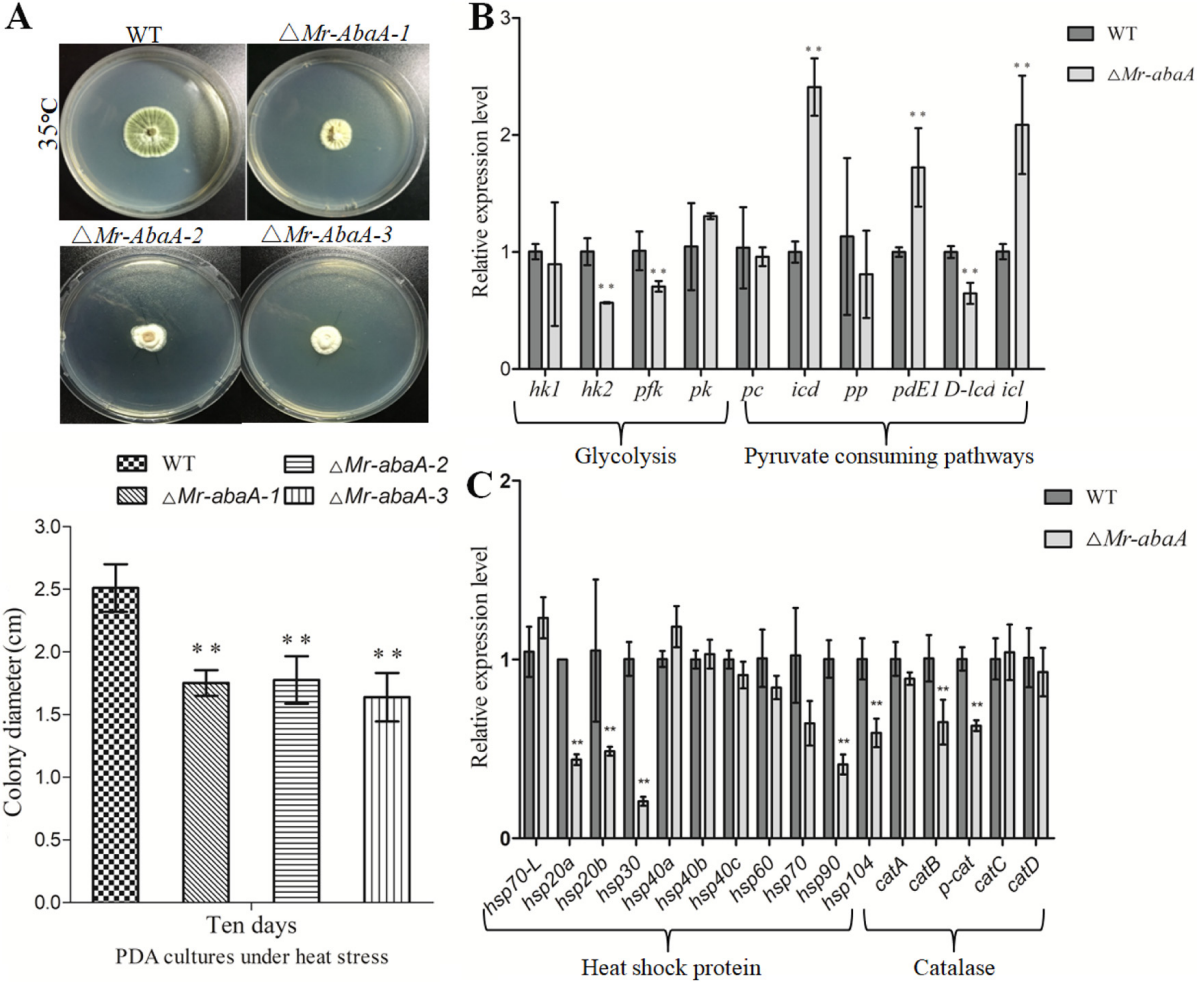
*Mr-abaA* is involved in heat tolerance. (A) Colony diameters of the WT strain and three *Mr-abaA* mutant hyphae on PDA plates with heat stress (35°C). (B and C) qRT-PCR analysis of the expression levels of heat-stress-responsive genes between WT and Δ*Mr-abaA* strains. **, *P < *0.01, Tukey’s HSD tests. Error bars in panels A and B indicate standard deviations of the means from three independent replicates.

To further investigate the role of *Mr-abaA* in heat tolerance, the growth of WT and mutant colonies was analyzed under heat stress because of the absence of conidia in the Δ*Mr-abaA* mutant. Intriguingly, compared with the WT strain, the mean colony diameters of three Δ*Mr-abaA* isolates were reduced by 30% ± 3%, 29% ± 3%, and 35% ± 4% under 35°C heat stress (Tukey’s HSD tests, *P < *0.01 [*n *= 3]) (Fig. 5A). Therefore, the Δ*Mr-abaA* mutant showed significantly increased sensitivity to heat stress. Furthermore, some key heat-stress-responsive genes were assessed for their transcript levels in the Δ*Mr-abaA* mutant, relative to the WT strain (32, 33). qRT-PCR results indicated that the heat stress significantly downregulated 2 of 4 genes involved in the glycolytic pathway, 5 of 11 genes encoding heat shock proteins, and 2 of 5 catalase genes but remarkably upregulated 3 of 6 genes involved in the pyruvate-consuming pathway in the Δ*Mr-abaA* mutant (Fig. 5B and 5C).

The rate of Δ*Mr-abaA* growth inhibitions in the presence of H_2_O_2_, Congo red, and NaCl was not different from that of the WT strain (see Fig. S2B). These results suggested that *Mr-abaA* is important for hyphal heat tolerance but is not involved in fungal antioxidant capacity, cell wall integrity, or osmotic stress.

### Mr-AbaA regulates *Mr-veA* expression by directly binding to its promoters.

The An-AbaA transcription factor recognizes specific DNA motifs called *abaA*-response elements (AREs), which are characterized by a CATTCY sequence (15). Additionally, it was reported that *abaA* interacts not only with the AREs in the *wetA* promoter region but also with AREs in the *velB* and *vosA* promoter regions (22). A previous study showed that MAA-05862 was named *velvet* family gene *vosA* and has already been knocked out in M. robertsii (26). In this study, a BLAST search was conducted to identify genes potentially coding for transcriptional regulators of the *velvet* family in the M. robertsii genome database, with the A. nidulans *velvet* family gene *veA* as a query. The results showed that four *velvet* orthologs were found in the fungal genome. Further phylogenetic analysis indicated that MAA-01811, MAA-00244, MAA-01976, and MAA-05862 were designated *Mr-veA*, *Mr-velB*, *Mr-vosA*, and *Mr-velC*, respectively (see Fig. S3).

**FIG 6:**
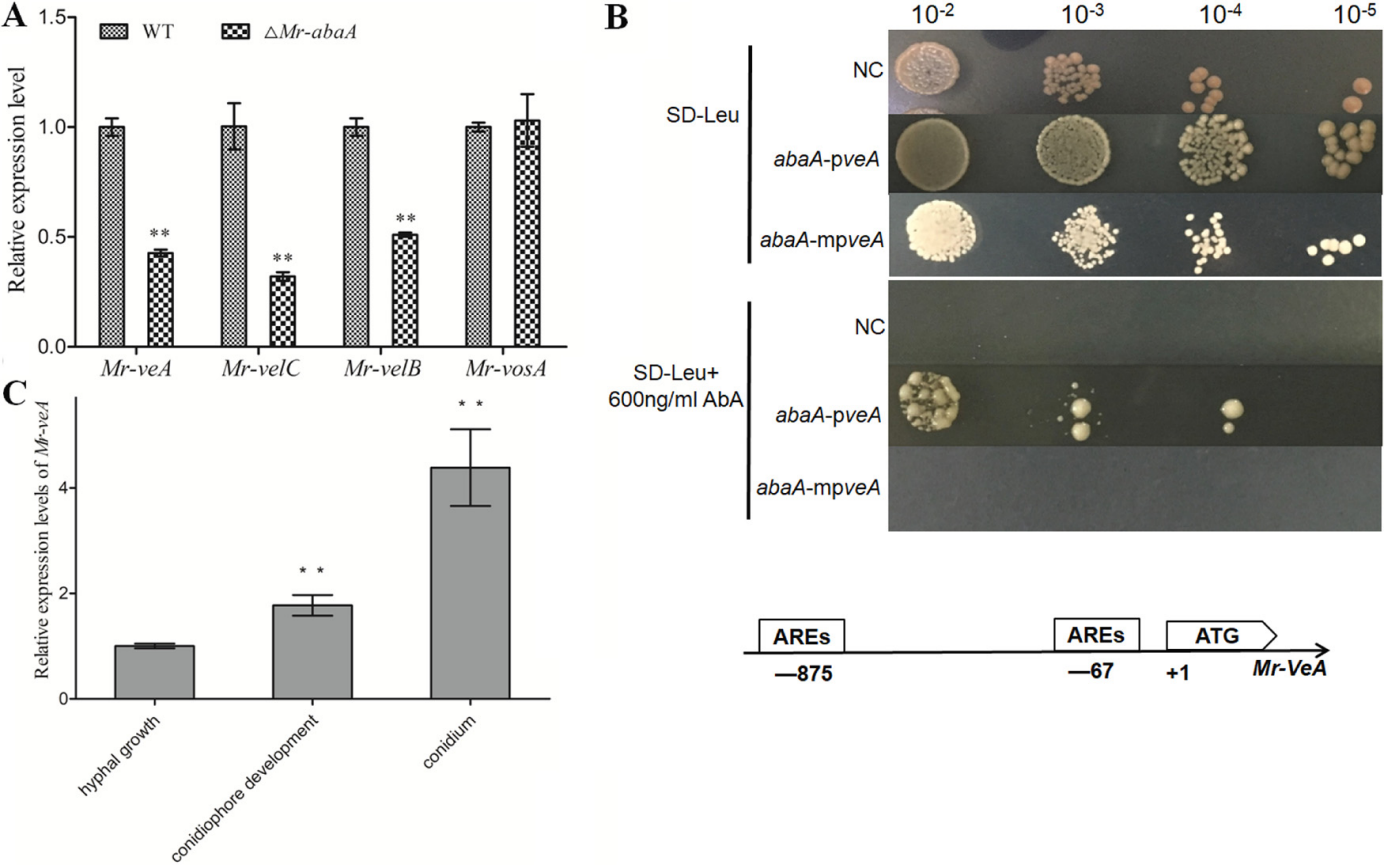
Interaction of Mr-AbaA with the promoter region of *Mr-veA*. (A) qRT-PCR analysis of the expression levels of *velvet* family genes in the WT strain and the Δ*Mr-abaA* strain. (B) Yeast one-hybrid assay to test the interactions of Mr-AbaA with the *Mr-veA* promoter regions. Yeast cells were transformed with both the pGADT7 AD vector containing the sequence of Mr-AbaA and plasmid pAbAi containing the *Mr-veA* promoter regions and mutated *Mr-veA* promoter regions. Transformed yeast cells were grown on SD-Leu medium with 600 ng/ml AbA, showing the interaction between the protein and the promoter region. NC, negative control; *abaA*-p*veA*, interaction between Mr-AbaA and the *Mr-veA* promoter region; *abaA*-mp*veA*, interaction between Mr-AbaA and the mutated *Mr-veA* promoter region; AbA, aureobasidin A. Putative ARE binding motifs are in the promoter regions of *Mr-veA* and *Mr-wetA*. One thousand-base pair portions of the *Mr-veA* and *Mr-wetA* promoter regions were analyzed. (C) Relative transcript levels of *Mr-veA* in the WT cultures in three different developmental stages. **, *P < *0.01, Tukey’s HSD tests. Error bars in panels A and C indicate standard deviations of the means from three independent replicates.

To investigate whether Mr-AbaA binds to the promoter region of *velvet* orthologs, bioinformatic analysis was performed, expression levels of these genes were determined, and yeast (Saccharomyces cerevisiae) one-hybrid analyses were carried out. First, the qRT-PCR analysis showed that the expression levels of *Mr-veA*, *Mr-velB*, and *Mr-velC* but not the expression of *Mr-vosA* were significantly reduced in the Δ*Mr-abaA* mutant at the conidiophore development stage (Tukey’s HSD tests, *P < *0.01 [*n *= 3]) (Fig. 6A). Thus, we selected *Mr-veA*, *Mr-velB*, and *Mr-velC* as candidates for yeast one-hybrid analysis. The positive clones showed that Mr-AbaA could physically bind to the promoter region of *Mr-veA* (Fig. 6B). However, recognition of the *Mr-velC* promoter region by endogenous yeast transcription factors resulted in unsuccessful yeast one-hybrid analysis, while Mr-AbaA could not interact with the *Mr-velB* promoter region (see Fig. S4). Further analysis of the promoter regions of *Mr-veA*, *Mr-velB*, and *Mr-velC* showed that only the *Mr-veA* promoter region contained two CATTCY AREs (Fig. 6B). Consistent with data from the qRT-PCR analysis and promoter sequence analysis, the yeast one-hybrid assay showed that Mr-AbaA can interact with the *Mr-veA* promoter region.

### Roles of *Mr-veA* in conidiation.

**FIG 7:**
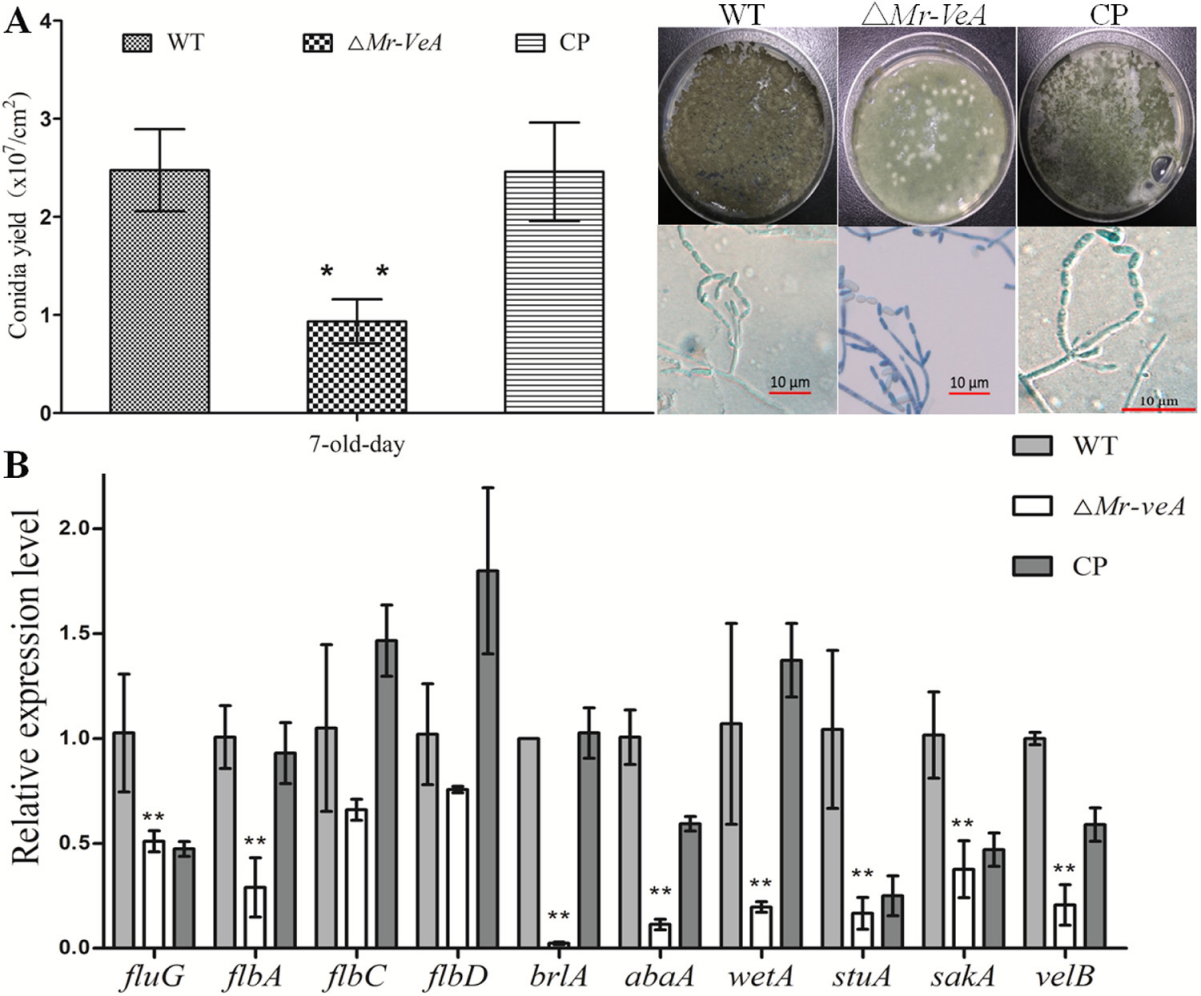
Functional evaluation of *Mr-veA* in conidiation. (A) Conidial yield evaluated by culturing the WT, Δ*Mr-veA*, and complementation (CP) strains on PDA plates at 7 dpi. Conidiophores were observed at the initial conidiation stage. (B) qRT-PCR analysis of the expression levels of conidiation-related genes in the WT, Δ*Mr-veA*, and complementation strains. **, *P < *0.01, Tukey’s HSD tests. Error bars in panels A and B indicate standard deviations of the means from three independent replicates.

To verify the roles of *Mr-veA* in conidiation, the transcriptional profiles of *Mr-veA* were monitored in the hyphal growth, conidiophore development, and conidium stages. The results showed that the transcript level of *Mr-veA* was increased in the conidiophore development and conidium stages, compared with the hyphal growth stage (Fig. 6C). For further study, a null *Mr-veA* mutant was constructed (see Fig. S1C). Conidial yields from 7-day-old cultures of the WT strain, the Δ*Mr-veA* mutant, and the complementation strain were quantified as 2.47(±0.42; *n *= 9) × 10^7^ conidia/cm^2^, 0.93(±0.23; *n *= 9) × 10^7^ conidia/cm^2^, and 2.46(±0.5; *n *= 9) × 10^7^ conidia/cm^2^, respectively, indicating a remarkable 62% reduction of conidial yield in the absence of *Mr-veA* (Tukey’s HSD tests, *P < *0.01 [*n *= 3]) (Fig. 7A). Nevertheless, microscopic examination showed that the null mutant of *Mr-veA* did not result in a distinctive defect in phialide formation during conidiation (Fig. 7A). Thus, deletion of *Mr-veA* repressed conidiation but did not alter the morphological pattern of asexual development, compared with the WT strain.

To further study the roles of *Mr-veA* in conidiation, the expression levels of conidiation-related genes in filamentous fungi were assessed in the null *Mr-veA* mutant. The qRT-PCR results demonstrated that the relative expression levels of genes, including upstream activators *flbG* and *flbA*, central regulators *brlA*, *abaA*, and *wetA*, or other conidiation-related genes (*stuA*, *sakA*, and *velB*) were significantly downregulated in the Δ*Mr-veA* mutant, compared with the WT strain and the complementation strain (Tukey’s HSD tests, *P < *0.01 [*n *= 3]) (Fig. 7B).

### Mr-AbaA regulates conidiation by interacting with the promoter regions of both *Mr-veA* and *Mr-wetA*.

**FIG 8:**
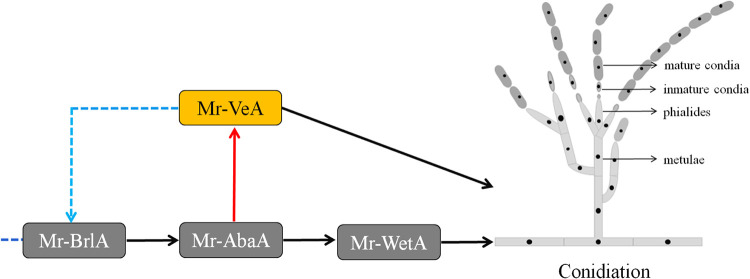
Putative regulatory model of the *Mr-abaA*-mediated regulation of conidiation in M. robertsii. Mr-AbaA positively regulated conidiation via *Mr-wetA* and *Mr-veA*, and the expression of *Mr-abaA* was activated by Mr-BrlA. The model shows hypothetical positive feedback control of conidiation involving Mr-AbaA, Mr-VeA, and Mr-BrlA. Solid arrows indicate positive regulation, and imaginary lines indicate uncharted regulation.

In our study, the data from the ARE search in the promoter region of *Mr-wetA*, the expression level of *Mr-wetA* in the Δ*Mr-abaA* mutant, and the yeast one-hybrid assay results indicated that Mr-AbaA physically binds to the promoter region of *Mr-wetA* (see Fig. S5), which is the same as in a previous report (26). In combination with the data from analysis of the interaction of Mr-AbaA with the *Mr-veA* promoter region and conidial characteristics in the *Mr-veA*-deleted strain, we concluded that Mr-AbaA regulates conidiation by interacting with the promoter regions of both *Mr-veA* and *Mr-wetA* (Fig. 8).

## DISCUSSION

The differentiation of functional phialides is critical for the conidiogenesis of filamentous fungi. After undergoing a period of vegetative growth, M. robertsii develops into functional phialides to produce conidia. Our study showed that Mr-AbaA is localized to the nuclei of phialides and that deletion of *Mr-abaA* results in abnormal phialides that produce aberrant abacus conidia. The null ability of the Δ*Mr-abaA* mutant to produce dark green conidia is responsible for the significant change in colony pigmentation. In another entomogenous fungus, B. bassiana, the Δ*abaA* mutant fails to generate clustered zigzag rachises (phialides) but cell clusters such as conidiation structures are infrequently present in old Δ*abaA* cultures (21). The *abaA* null mutant of A. nidulans forms aberrant conidiophores that fail to produce conidia (13). Similarly, deletion of *abaA* results in a defective phenotype similar to those of conidiophores in A. fumigatus, T. marneffei, P. digitatum, and F. graminearum (18–21). These findings suggest that *abaA* has conserved functions in the differentiation of conidiogenous structures in filamentous fungi, although the conidiation patterns in those fungi are different from each other.

A conidium is a pivotal unit for fungal survival, dispersal, and infection in the environment. The transcript level of *Mr-abaA* in mature conidia was significantly higher than that in other phases. Laser scanning confocal microscopy (LSCM) analysis also showed that Mr-AbaA is localized in the nucleus of mature conidia. Similar transcriptional profiles and subcellular localization of *abaA* were observed in F. graminearum (19). Therefore, the high level of expression of *abaA* in mature conidia indicated that *abaA* may play roles in conidial maturation in these filamentous fungi. However, the Δ*Mr-abaA* mutant failed to generate normal conidia and, as a result, we were not able to study conidial maturation. Therefore, further studies that apply RNA interference technology to repress the transcript level of *Mr-abaA* in conidia will contribute to understanding the function of *Mr-abaA* in conidial maturation.

The regulatory pathway of *abaA* has been extensively studied in A. nidulans. An-AbaA directly activates not only *wetA* in the late stage of conidiation but also *vosA* and *velB* in conidial maturation (3, 22). A previous study also proved that Mr-BlrA can upregulate *Mr-abaA*, which in turn regulates *Mr-wetA* during conidiation in M. robertsii (26). Transcriptional profiles showed that the transcript level of *Mr-wetA* was increased in the conidiophore development and conidium stages, compared with the hyphal growth stage (see Fig. S6 in the supplemental material). However, our results indicated that *Mr-abaA* regulated conidiation via the *velvet* family gene *Mr-veA*. Compared with the hyphal growth stage, *Mr-veA* is highly expressed in the conidiophore development and conidium stages, compared with the hyphal growth stage. Deletion of *Mr-veA* also significantly repressed conidiation. Therefore, we found that a *velvet* family gene, *veA*, was directly activated by Mr-AbaA to regulate conidiation.

In this study, deletion of *Mr-abaA* resulted in significant downregulation of conidiation-related genes, including *Mr-brlA* and *Mr-veA*. We further found that deletion of *Mr-veA* also resulted in downregulation of *Mr-brlA* and *Mr-abaA*. In Aspergillus niger, it has been demonstrated that *veA* affects conidiation by regulating *brlA* expression levels (34). Therefore, there is a possibility of positive feedback control of conidiation on *Mr-abaA* mediated by *Mr-veA* and *Mr-brlA*. Unfortunately, Mr-VeA could not directly bind to the promoter regions of *Mr-brlA*, *Mr-abaA*, *and* Mr-*wetA* in our yeast one-hybrid assays (see Fig. S7). In Aspergillus flavus, VeA, VelB, and LaeA form a heterotrimeric complex, and FluG, which is a gene upstream of *brlA*, is probably an interacting partner of VelB (35). Therefore, VeA may generate a velvet complex interacting with FluG to regulate *brlA* expression, and the detailed mechanism by which Mr-VeA regulates *Mr-brlA* remains to be studied in future investigations.

The dimorphic transition between hypha and hyphal body (also called blastospore) forms is an important phenomenon in dimorphic fungi (18, 21, 36). The process of dimorphic transition has been well studied in M. robertsii; however, the molecular mechanism involved remains poorly understood. Previous studies showed that MAD1 is an adhesion protein whose mutant suppressed blastospore formation in M. robertsii (37). Our results show that *Mr-abaA* is indispensable for the separation of blastospores in submerged culture, but the deletion of *Mr-abaA* did not affect the expression level of MAD1 (see Fig. S8). T. marneffei with deletion of *abaA* fails to switch correctly from filamentous to yeast-like cells, and the B. bassiana Δ*abaA* mutant does not produce blastospores (18, 21). These results indicate that the AbaA function is conserved in these dimorphic fungi.

In conclusion, bioinformatic analysis and data on the transcriptional profiles and subcellular localization of Mr-AbaA indicated that AbaA functioned in the conidiophore development and conidium stages as a transcription factor. Microscopic examination showed that the *Mr-abaA* mutant differentiated into defective phialides to produce abacus structures instead of conidia. In addition, *Mr-abaA* is required for both aerial conidiation and submerged blastospore separation *in vitro*. Moreover, Mr-AbaA regulates conidiation by interacting with the promoter regions of *Mr-veA* and *Mr-wetA*. This finding provides new insight into the regulation of conidiation of this important entomopathogenic fungus.

## MATERIALS AND METHODS

### Strains and culture conditions.

The WT M. robertsii strain ARSEF 23 (ATCC number MYA-3075) was cultured on PDA (20% potato, 2% dextrose, and 2% agar [wt/vol]) in the dark at 25°C for 10 days to produce conidia. For liquid incubation, fungal strains were grown in PPD medium (20% potato, 2% dextrose, and 1% peptone [wt/vol]) and SDY medium (4% glucose, 1% peptone, and 1% yeast extract) at 25°C on a rotary shaker. The Y1H strain was used for yeast one-hybrid tests. Yeast cells were grown on yeast-peptone-dextrose agar (YPDA) (1% yeast extract, 2% peptone, 2% dextrose, adenine hemisulfate, and 1.5% agar), yeast-peptone-dextrose (YPD) medium (1% yeast extract, 2% peptone, and 2% dextrose), or synthetic dropout (SD) agar medium. Agrobacterium tumefaciens strain AGL-1 was cultured on solid yeast extract-beef (YEB) medium (0.5% sucrose, 1% tryptone, 0.1% yeast extract, 0.05% MgSO_4_·7H_2_O, and 1.5% agar [wt/vol]) at 28°C.

### Transcriptional profiling of *Mr-abaA* and protein localization.

The WT strain was cultured on PDA for 10 days at 25°C in the dark and spread with 100-μl aliquots of a suspension of 10^7^ conidia/ml. Total RNAs were extracted from samples that had been separately collected at time points of 36 h (hyphal growth), 72 h (conidiophore development), and 240 h (conidium stage) after inoculation using TRIzon reagent (Cwbio, Hefei, China). Then, RNA was reverse transcribed into cDNA using a ReverTra Ace qPCR RT master mix with genomic DNA (gDNA) remover kit (Toyobo, Japan). Three of the cDNA samples were used to assess the transcript levels of *Mr-abaA* via qRT-PCR with the CFX96 RT-PCR system (Bio-Rad, USA). The fungal glyceraldehyde 3-phosphate dehydrogenase (GAPDH) gene was used as a standard gene. The 2^−ΔΔ^*^CT^* method was used to calculate the relative gene transcript levels (38).

To construct the AbaA::EGFP fusion protein, the full sequence of *Mr-abaA* with an upstream ∼1,000-bp fragment was amplified and cloned with the full sequence of EGFP into the pDHt-SK-*bar* vector, with which WT cells were transformed. Each transgenic strain was cultured on PDA for initial and full conidiation at 25°C in the dark. Mature conidia and hyphal cells were stained with the nucleus-specific dye DAPI and were then observed for subcellular localization under LSCM.

### Phylogenetic analysis of *abaA* and *veA* in different fungi.

The Aspergillus nidulans FGSC A4 AbaA (GenBank accession number XP_658026) and VeA (GenBank accession number XP_658656.1) proteins were used as queries to search the M. robertsii genome available in the NCBI database via online BLASTP analysis (https://blast.ncbi.nlm.nih.gov/Blast.cgi). Amino acid sequences of *abaA* homologs in the genomic databases for M. robertsii, A. nidulans, A. flavus, *Talaromyces marneffei*, Claviceps purpurea, P. lilacinum, B. bassiana, F. graminearum, N. crassa, Magnaporthe oryzae, *Candidaglabrata*, Naumovozyma castellii, and Saccharomyces cerevisiae were downloaded from the NCBI database (http://ncbi.nlm.nih.gov). Phylogenetic analysis was conducted using MEGA6 software (http://www.megasoftware.net). The NLS motif of Mr-AbaA was predicted online at NLStradamus (http://www.moseslab.csb.utoronto.ca/NLStradamus).

### Generation of *Mr-abaA* and *Mr-veA* mutants.

Deletions of *Mr-abaA* and *Mr-veA* were performed based on homologous recombination, as we described previously (39). Briefly, the 5′-flanking region (BamHI) and 3′-flanking region (XbaI) of the genes were amplified from gDNA by PCR and cloned onto the binary vector pDHt-SK-*bar* (conferring resistance to glufosinate ammonium) to construct the deletion mutant using Agrobacterium tumefaciens-mediated transformation. The integration event was verified by PCR and RT-PCR. The primers used in this study are listed in Table S1 in the supplemental material.

### Mutant phenotype assays.

For the growth assay, hyphal blocks (4-mm diameter) were obtained from the WT and Δ*Mr-abaA* strains grown on cellophane-overlaid SDAY medium for 4 days and attached centrally to PDA, SDAY, and 1/4 SDAY (amended with one-quarter of the nutrients of SDAY) plates. At 10 days postinoculation (dpi), all colony diameters were measured as indices of radial growth rates, using the cross-crossing method (40).

For chemical stress tolerance assays, hyphal blocks (4-mm diameter) of WT and three Δ*Mr-abaA* strains were attached in the center of PDA plates with supplementary chemical reagents, including the cell wall-disturbing compound Congo red (2 mg/ml), H_2_O_2_ (5 mM) as an inducer of oxidative stress, and NaCl (0.5 M) as an inducer of osmotic stress. To investigate the fungal hypha responses to heat stress, hyphal blocks (4-mm diameter) were attached centrally to PDA plates and cultured at 35°C for 10 days, and all colony diameters were measured (32). The rate of growth inhibition (RGI) was calculated as (*C* − *S*)/*C* × 100, where *C* is the growth rate of the control and *S* is the growth rate under stress conditions (39).

To assay the conidiation capacity of the WT strain and each mutant, 30 μl of a suspension of 10^6^ conidia/ml was evenly spread on PDA plates (6-mm diameter) and cultured in the dark at 25°C for 7 and 14 days. On 7 and 14 dpi, fresh conidia from the WT strain and each mutant were separately collected into 30 ml of 0.05% Tween 80, and conidia were dispersed by vibration. The concentration of conidial suspensions was measured using a hemocytometer and then converted to the number of conidia produced per unit area (square centimeter) of plate culture. Because the Δ*Mr-abaA* mutant failed to produce conidia, its conidiation capacity was completely lost. During the culture period, the sporulation states of each strain were observed under a microscope.

For qRT-PCR analysis, each strain was cultured on PDA plates for 2.5 days, and the hyphae were collected for total RNA extraction to conduct conidiation-related gene expression analysis. qRT-PCR analysis was performed using the qPCR SYBR green master mix (Vazyme, China). Primers for qRT-PCR are listed in Table S2 in the supplemental material.

Assessment of blastospore formation was performed as described previously (21). Briefly, submerged cultures of the Δ*Mr-abaA* and WT strains were initiated with hyphal blocks (4-mm diameter) cultured in SDY broth. After 3 days of culture, collected hyphae were rinsed twice with sterile water and filtered through lens-cleaning tissues to remove resuspended blastospores. All of the aliquots in flasks were standardized to a final concentration of fresh hyphal mass of 1 mg/ml and were incubated in SDY broth and PPD broth for 4 days with shaking (130 rpm). The blastospore concentration was assessed from each sample using a hemocytometer and was used to compute the absolute blastospore yield (number of blastospores per milliliter) in each submerged culture.

The aforementioned data from the experiments with three replicates were subjected to one-way analysis of variance, followed by Tukey’s HSD test for phenotypic changes among the tested fungal strains.

### Yeast one-hybrid assay.

The *Mr-veA* target promoter region with an ∼615-bp DNA fragment, the mutated AREs (CttaCC) in the *Mr-veA* target promoter region with an ∼615-bp DNA fragment, and the *Mr-wetA* target promoter region with an ∼645-bp DNA fragment were amplified and cloned into the linearized pAbAi vector (Clontech, USA). The plasmids (pAbAi-p*veA*, pAbAi-mp*veA*, and pAbAi-p*wetA*) were linearized and cloned into Saccharomyces cerevisiae Y1HGold cells (Clontech). Transformed strains were grown on SD-Ura agar medium. Subsequently, the Mr-AbaA coding region was amplified from cDNA and cloned into the linearized pGADT7-AD vector (Clontech). The recombinant plasmid pGADT7-*abaA* was further transformed into the Y1H+baitGold (pAbAi-p*veA*, pAbAi-mp*veA*, and pAbAi-p*wetA*) strain. The transformed cells were plated on an SD-Leu agar medium with 600 ng/ml aureobasidin A to identify the interactions of Mr-AbaA with the *Mr-veA* and *Mr-wetA* promoters. Y1HGold (pAbAi-p*veA*, pAbAi-mp*veA*, and pAbAi-p*wetA* plus pGADT7-AD) cells were used as a negative control.
