# Supplementary material for: Mr-AbaA Regulates Conidiation by Interacting with the Promoter Regions of Both Mr-veA and Mr-wetA in Metarhizium robertsii
Source: Microbiol Spectr. 2021 Sep 8;9(2):e00823-21. doi: 10.1128/Spectrum.00823-21 (PMC8557821; doi:10.1128/Spectrum.00823-21)
Supplement: SUPPLEMENTAL FILE 1 — Supplemental material. Download SPECTRUM00823-21_Supp_1_seq12.pdf, PDF file, 0.7 MB. [file spectrum00823-21_supp_1_seq12.pdf]

SUPPLEMENTAL MATERIAL

Mr-AbaA regulates conidiation by interacting with the promoter region of *Mr-veA* and *Mr-wetA* in *Metarhizium robertsii*

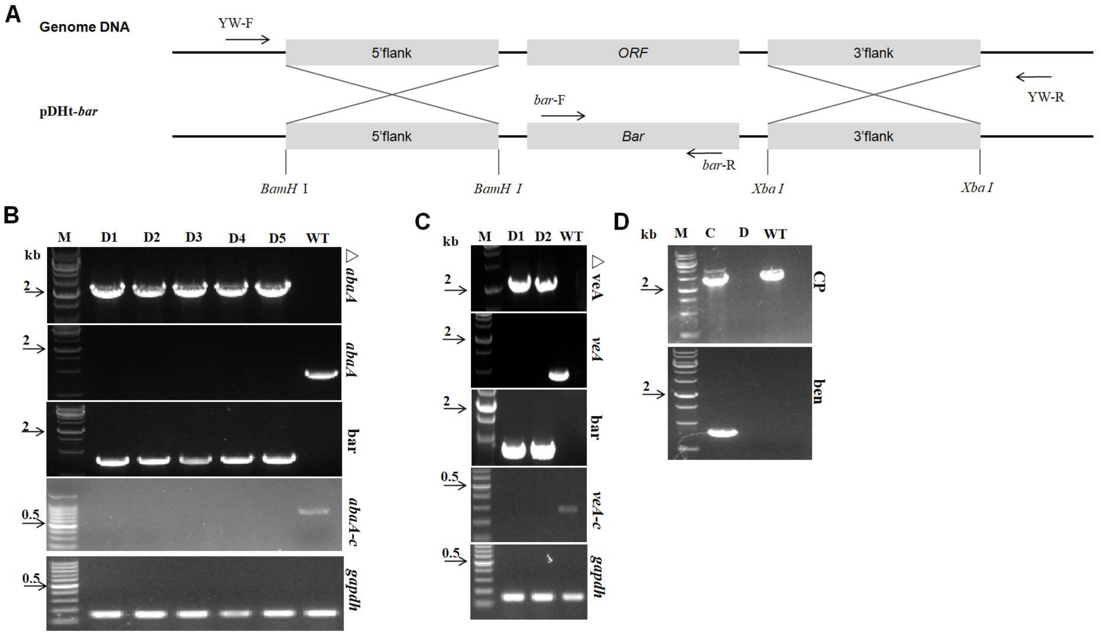

Figure S1.Gene deletions of *Mr-abaA*, and *Mr-veA* in *M. robertsii* ARSEF 23. (A) Schematic diagram of gene deletions by the homologous recombination approach. **(B, C, D)** Confirmation of the *Mr-abaA*, *Mr-veA* and the complemented strain of *Mr-veA* via PCR and RT-PCR. The targeted genes and *gapdh* were amplified using DNA from a different strain or cDNA as templates, respectively. M: marker. D: gene deletion mutant; WT: the wild-type strain. C: complemented strains

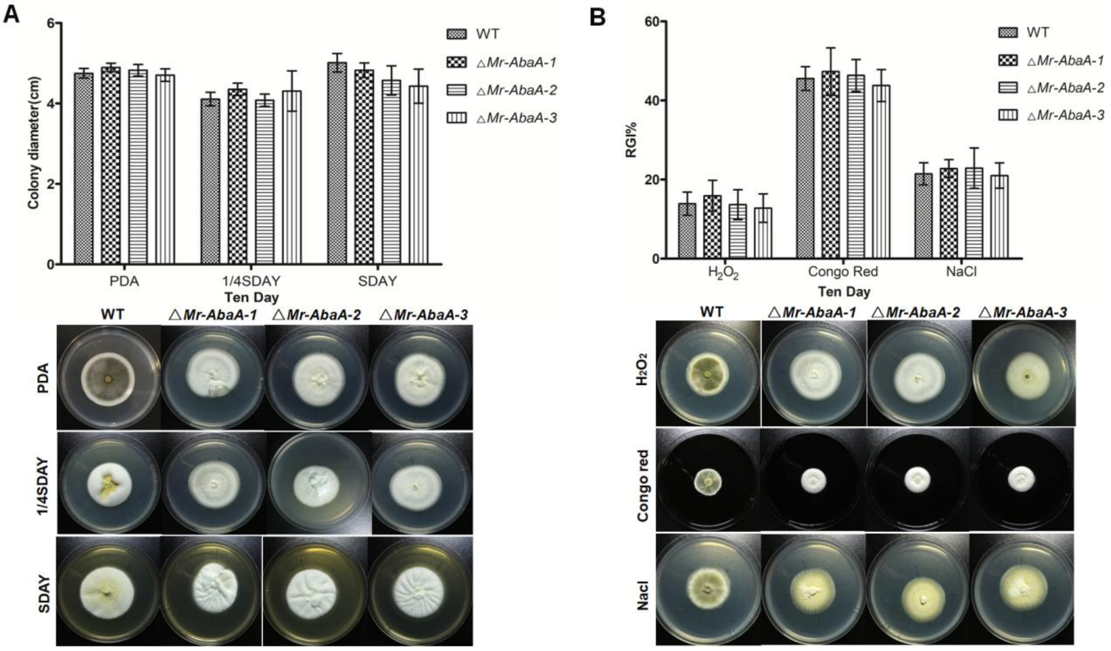

Figure S2. Effects of *Mr-abaA* deletion on hyphal growth of *M. robertsii* on different media. (A) The colony diameters of each strain were measured after 10 dpi on PDA, SDAY, and 1/4 SDAY plates. The colony phenotype of each strain cultured for 10 days on the above medium was evaluated. **(B)** Relative growth inhibition (RGI) of each strain fungal colony after 10 days of culture under H_2O_2, Congo red, and NaCl stress on PDA. The colony phenotype of each strain cultured under the above stress conditions for 10 days was evaluated.

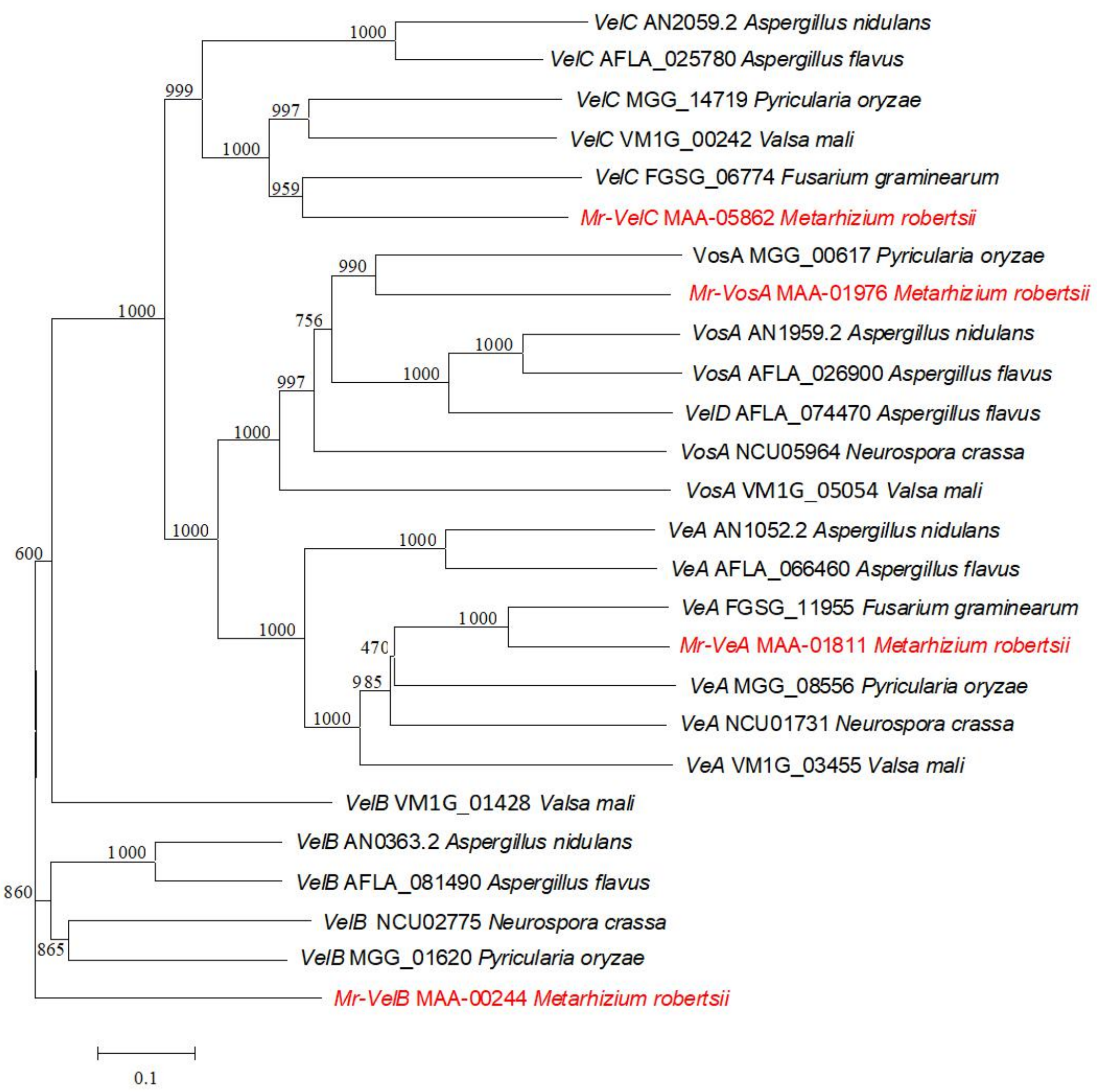

Figure S3. **Phylogenetic tree analysis of the *M. robertsii velvet* family genes.**

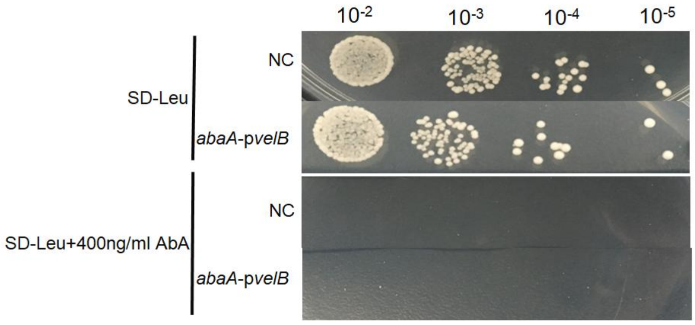

Figure S4. **Analysis of the protein-DNA interaction.** Yeast one-hybrid assay to test the interactions of Mr-AbaA with the *Mr-velB* promoter regions NC: Negative control. *abaA*-p*velB*: interaction between the Mr-AbaA protein and the *Mr-velB* promoter region. AbA, aureobasidin A.

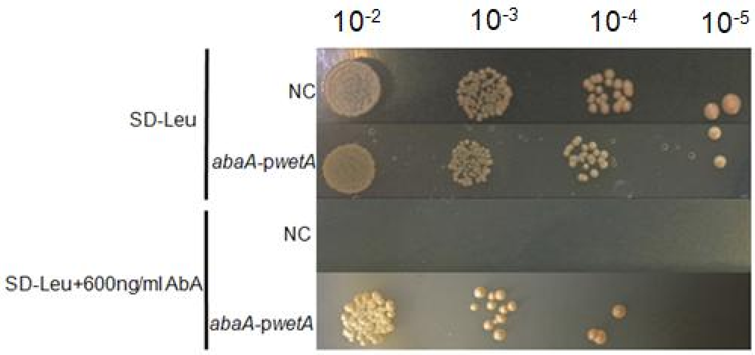

Figure S5. **Analysis of the protein-DNA interaction.** Yeast one-hybrid assay to test the interactions of Mr-AbaA with the *Mr-wetA* promoter regions NC: Negative control. *abaA*-p*wetA*: interaction between the Mr-AbaA protein and the *Mr-wetA* promoter region. AbA, aureobasidin A.

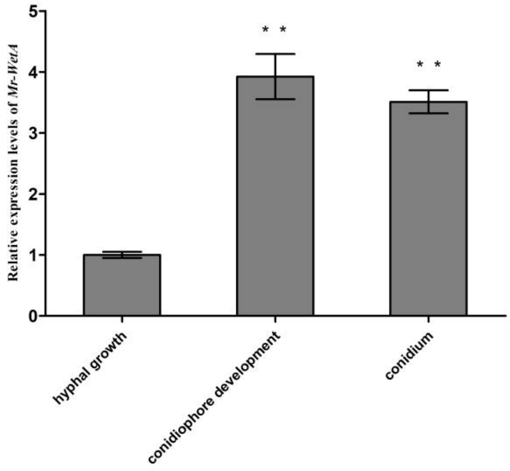

Figure S6. Transcriptional profiles of *Mr-wetA*.

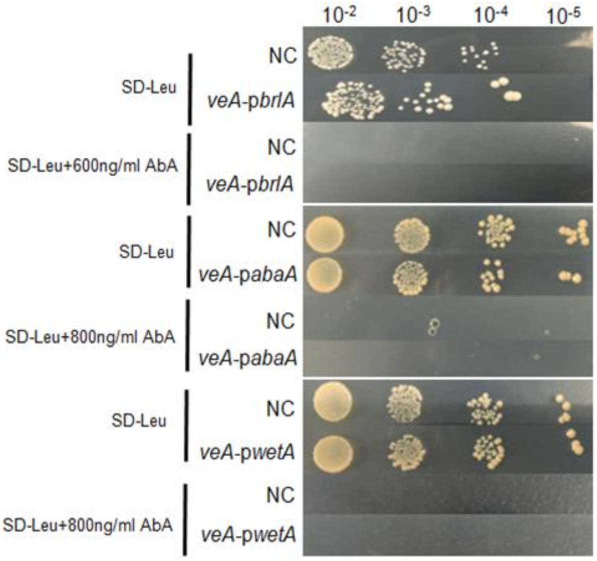

Figure S7. **Analysis of the protein-DNA interaction.** Yeast one-hybrid assay to test the interactions of Mr-VeA with the *Mr-brlA*, *Mr-abaA*, and *Mr-wetA* promoter regions. NC: Negative control. *veA*-p*brlA*: interaction between the Mr-VeA protein and the *Mr-brlA* promoter region. *veA*-p*abaA*: interaction between the Mr-VeA protein and the *Mr-abaA* promoter region. *veA*-p*wetA*: interaction between the Mr-VeA protein and the *Mr-wetA* promoter region. AbA, aureobasidin A. The results showed that the Mr-VeA could not directly bind to the promoter regions of *Mr-brlA*, *Mr-abaA*, and *Mr-wetA*.

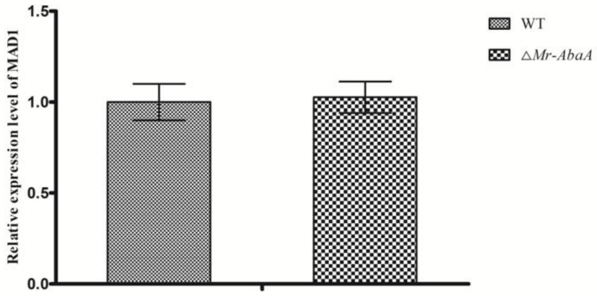

Figure S8. **qRT-PCR analysis of the expression level of MAD1 between the WT and Δ*Mr-abaA*.**

Table S1 Primers used for functional analyses of two genes in *M. robertsii*.

Primer name	Primer sequence(5'- 3')[a]	Purpose of use
**Component of *Mr-abaA***		
abaA-SF	CGGGATCCTCTCAAGGCAATGGATGGGC	
abaA-SR	CGGGATCCGACGCAACGAACGAGAACCA	Amplifying upstream flanking sequence
abaA-XF	GCTCTAGATTATCTCCACGCCCTCCT	
abaA-XR	GCTCTAGATTTGAAATATCCGGTCCTTT	Amplifying downstream flanking sequence
abaA-MF	AGCACCATCTATGCAAACCA	
abaA-MR	GTAGGCAACATCATCCCGAA	PCR for *abaA* to screen the transformants
abaA-YWR	TCTTGTGCGGTGCGGATAG	PCR for △*abaA* to confirm the mutant
abaA-GFP-b1F	ttcctgcagcccgggggatccAAGAAATCGAGAAGATGCAGCG	Amplifying the promoter region and
abaA-GFP-b1R	tgctcaccatCCATCCAGCAGTGACGGGC	*Mr-abaA* sequence to construct the EGFP
abaA-GFP-b2F	tgctggatggATGGTGAGCAAGGGCGAGG	fusion protein
abaA-GFP-b2R	tcatcttctgtcgacggatccTTACTTGTACAGCTCGTCCATGCC	Amplifying EGFP sequence
abaA-cdsF	gccatggaggccagtgaattcATGTCTTCACTCTTTCAGCCAAGA	Amplifying the *Mr-abaA* ORF for yeast
abaA-cdsR	atgcccacccgggtggaattcTCACCATCCAGCAGTGACGG	one-hybrid test
p-*wetA*-F	aattcgagctcggtacccgggATGTTTGAGCGGTAATGGC	
p-*wetA*-R	cgaggtcgacagatccccgggGGCGTGTGATGTTGTGTGT	Amplifying *Mr-wetA* promoter region
p-*veA*-F	aattcgagctcggtacccgggTGAGTTGATCGCCACCCTGC	
p-*veA*-R	cgaggtcgacagatccccgggTTTGTCGTAGCAGTCTCAAGGTTG	Amplifying *Mr-veA* promoter region
p-*velB*-F	aattcgagctcggtacccgggTTGTGATTGACTCTTTGCGA	
p-*velB*-R	cgaggtcgacagatccccgggGTTCAAATGGTGGTTTCTGG	Amplifying *Mr-velB* promoter region
abaA-F	AGCACCATCTATGCAAACCA	
abaA-R	GTAGGCAACATCATCCCGAA	RT-PCR for *abaA*-c to confirm the mutant
**Component of *Mr-VeA***		
veA-SF	CGGAATTCCCCCCTACTTGCGATACGA	
veA-SR	CGGGATCCGCGAATGAACGGGATGGTG	Amplifying upstream flanking sequence
veA-XF	GGACTAGTTCGCTCTGCTACTTTGCCTA	
veA-XR	GGACTAGTCGCTCCACTTTTTCCTTCTC	Amplifying downstream flanking sequence
veA-MF	GACTACCAAGGAGGAAAAAGAC	
veA-MR	ATAATAGGAGACACAGGCACAG	PCR for *Mr-veA* to screen the transformants
veA-YWR	GCGGGAAAGGAGAAAATAGTT	PCR for △*Mr-veA* to confirm the mutant
veA-F	TGAAAGCCAATAGCGACAGAC	
veA-R	CGGGAAAGATGAAGTAACCAG	RT-PCR for *Mr-veA*-c to confirm the mutant
veA-cdsF	gccatggaggccagtgaattcATGGCCGCCTCATCTGTACA	Amplifying the *Mr-veA* ORF for yeast
veA-cdsR	atgcccacccgggtggaattcCTATTGGTACCGGTTGAACTGAATC	one-hybrid test
p-*brlA*-F	aattcgagctcggtacccgggGCCTCAACTTCACCATACATGGG	
p-*brlA*-R	cgaggtcgacagatccccgggGTGTTGTTATTGTTGCTGTTGCTG	Amplifying *Mr-brlA* promoter region
p-*abaA*-F	aattcgagctcggtacccgggCTGGGCGCTGTGTAGATAGATCT	
p-*abaA*-R	cgaggtcgacagatccccgggAACTGCAAAGGCAAAAAAAAGA	Amplifying *Mr-abaA* promoter region
veA-CPF	aagcttcgcactagttctagaTTGATCGCCACCCTGCAT	
veA-CPR	cgcggtggcggccgctctagaGACGCCGACTCCTTCCATG	PCR for CP to screen the transformants
ben-F	CCTCCACATAGACCCGTGTTCGT	

ben-R	CAGAGGAGCCTGAATGTTGAGTG	PCR for CP to screen the transformants
bar-F	ATTTTGGTTTAGTCGTCCAGGCG	
bar-R	AGCTGCCAGAAACCCACGTCATG	PCR for mutants to screen the transformants

a: The lowercase letter in primers are required for cloning the PCR products into plasmids by homologous recombination and the red font showed the restriction enzyme.

Table S2 Primers used for qRT-PCR analysis

Gene	Tag locus	Annotation	primer (5'- 3')
gapdh	MAA_07675	glyceraldehyde 3-phosphate dehydrogenase	GACTGCCCGCATTGAGAAG/AGATGGAGGAGTTGGTGTTG
fluG	MAA_00122	protein fluG	TGCGGGTTGAATACGG/ CTCCACCTCTTTCTCCTTGA
flbA	MAA_06313	developmental regulator flbA	ACTCCAAAGGGCATCACG/ CAACAAAGCGGCGGAATA
flbC	MAA_04342	developmental regulator flbC	TTTCCAATCTACGACGACA/ AGTCCGATTGATGGTCTTC
flbD	MAA_03655	developmental regulator flbD	AACGATGGGCTGAGATTG/ GGTGATTGAGTTTCGGATG
brlA	MAA_10599	central regulator BrlA	ATCCTTTTGCTACTCACCA/ ACCATTCCATCCAACTTCT
abaA	MAA_00694	central regulator AbaA	AAACCACTATTCCTGCTCC/AGCCTGCCTGTTACGATA
wetA	MAA_02845	central regulator WetA	CGACGAAATAGGAAAGCA/ TGAAGTGGAGGAGATACGG
stuA	MAA_02988	APSES transcription factor StuA	GCAAGGCACCAACCCACT/ TGCTGCTCCGTAGGCTGA
veA	MAA_01811	velvet family gene VeA	TGAAAGCCAATAGCGACAGAC/CGGGAAAGATGAAGTAACCAG
velB	MAA_00244	velvet family gene velB	CAGGGAAACCTCTTGGGA/ TCGCCGAAACCGCACATT
vosA	MAA_01976	velvet family gene vosA	TTCACCATCACTCGGCACC/ CTTTTTCCTTTCCAACGGC
velC	MAA_05862	velvet family gene velC	AGACGGGTTCAAATAGCC/ TGTTTACTGAGGACGGAT
hk1	MAA_04209	Hexokinase	ATTCAAAGAACGTGGTGCCC/ATTGCCAGCCTTGACTCTCT
hk2	MAA_05057	Hexokinase	GTCACTTCAAGCCGCTACAG / CAACTCGCGAATCTCTGACG
pfk	MAA_09075	Phosphofructokinase	TTGCCAACATTATCCGCGAG / CTTCTTGACGCGGTTATGGG
pk	MAA_06851	Pyruvate kinase	TGTCTGGTGAAACGGCAAAG / CAATGATACCACCAGCAGCC
pc	MAA_04366	Pyruvate carboxylase	TCGGGAGATATGCTGAACCC/ ACACCAGTACCAGCAGAGTC
icd	MAA_03118	Isocitrate dehydrogenase	TAAGAATCCGGTCGTGGAAC/ GTGACCTGGTCGTTGGTCTT
pp	MAA_02781	Pyruvate permease	TCATCCCCATCCACCTCATG/ TAGACGCAGCCCATGAAGAT
pdE1	MAA_08787	Pyruvate dehydrogenase E1 component beta subunit	GTCAACTCTGCCGGAAAGAC/ CAGCCTTGAGAAGACCCTTG
D-lcd	MAA_07000	D-lactate dehydrogenase	TGCCTACGACCCATTTCCAT/ TATTCACCAGCATCACCCCA
icl	MAA_04402	Isocitrate lyase	CCGTCATGAAGCTCACCAAG/ AGGTCCGTGCCCATAATGTC
hsp70-L	MAA_03832	Hsp70-like protein	AGGTCCTGCAGCTCATTTCT/ CCGGCAAACTCGTAGTCATG
hsp20a	MAA_10381	Heat shock protein Hsp20	CGCCCAAATAAAGGAGCCAG/ CGCCCAAATAAAGGAGCCAG
hsp20b	MAA_07190	Heat shock protein Hsp20	ACATTGAATTCACCGAGCCG/ CAGTACTTGTACTTGGGCGC
hsp30	MAA_04014	Heat shock protein 30	CTGAGCCCGAGGAGAAG/ GACACGGTTGGGAAAGT
hsp40a	MAA_02497	Heat shock protein 40	AGCGAGGCGAGGTGAA/ GGTGGTGGCAGCATTT
hsp40b	MAA_03231	Heat shock protein 40	AGAGGAAGCCAACGACG/ TCCGCAAGAGTAACAACGAG
hsp40c	MAA_06393	Heat shock protein 40	CGCCAACAGGAAAGACT/ GCCGTGGTAAGAGGAAT
hsp60	MAA_07685	Heat shock protein 60	CGGCCAACTTTGACCAG/ CAATGACAACGGAACCCT
hsp70	MAA_00810	Heat shock protein 70	TTCAAAGAGGGATACCACC/ TTCAAAGAGGGATACCACC
hsp90	MAA_04726	Heat shock protein 90	TATGTCCGCCGTGTCTT/ TGTTCTGCTGGAGGGTC
hsp104	MAA_03534	Heat shock protein 104	TCTGCGGTCCCTCTGGT/ CGGCTAAGTGCGTGTCG
catA	MAA_05879	Catalase	ATCAACAGACCCGTTGTTCC/ GTGAAGAAGCCTTTGCCTTG
catB	MAA_06740	chanoclavine synthase catalase protein	TCCACTGGTTCCTGTCATCC/ GCATCTTCGGGGTCAATGAC
p-cat	MAA_10327	Peroxisomal catalase	CGATATTGGGGCACTGAAGC/ ACGGGTTCCAGATCTCCTTG
catC	MAA_01261	Catalase	ATTGCCCATTCCTTCAACGG/ CTGCCTGTTCAAAATCCGCT
catD	MAA_05785	Catalase	GAGATGGTGGCATGGTTGTC/ CGTCACATCGCCAAAGAGAG
